# 肺结节精准管理专家共识（2026年版）

**DOI:** 10.3779/j.issn.1009-3419.2026.102.08

**Published:** 2026-05-20

**Authors:** Juan ZHOU, Yudong ZHANG, Qinghua ZHOU, Chang CHEN, Chunxia SU, 肺结节精准管理专家共识（2026年版)编写委员会

**Affiliations:** 1 200082 上海，同济大学附属上海市肺科医院肿瘤综合诊治融合病房; 1 Comprehensive Diagnosis and Treatment Integration Ward for Tumors, Shanghai Pulmonary Hospital Affiliated to Tongji University, Shanghai 200082, China; 2 510120 广州，广州医科大学附属第一医院胸外科; 2 Department of Thoracic Surgery, The First Affiliated Hospital of Guangzhou Medical University,Guangzhou 510120, China; 3 610041 成都，四川大学华西医院肺癌中心; 3 Lung Cancer Center, West China Hospital, Sichuan University, Chengdu 610041, China; 4 200082 上海，同济大学附属上海市肺科医院胸外科; 4 Department of Thoracic Surgery, Shanghai Pulmonary Hospital Affiliated to Tongji University, Shanghai 200082, China

**Keywords:** 肺肿瘤, 筛查, 低剂量螺旋计算机断层扫描, 精准管理, 随访策略, 专家共识, Lung neoplasms, Screening, Low-dose spiral computed tomography, Precise management, Follow-up strategy, Expert consensus

## Abstract

随着低剂量螺旋计算机断层扫描的广泛应用和影像技术的进步，肺结节的检出率显著提高，但传统“一刀切”的管理策略已难以满足精准诊疗需求。本专家共识由上海市肺科医院苏春霞教授团队和陈昶教授团队共同发起，联合肿瘤内科、呼吸内科、胸外科、介入科、影像科、病理科等多学科专家共同制定，旨在针对现有指南关注较少或存在分歧的临床争议问题，提供科学、实用的决策参考。共识采用GRADE方法进行证据评估，通过德尔菲法2轮问卷调查形成18条推荐意见，聚焦特殊人群差异化筛查、人工智能辅助诊断、机器人辅助穿刺、分子标志物应用、低风险结节主动监测、侵入性干预时机、随访终止条件、多发结节管理策略以及早期肺癌个体化淋巴结清扫范围和手术方式选择等核心问题，为肺结节从筛查到治疗的全流程精准管理提供补充性指导意见，以期在提升肺癌患者长期生存率的同时，最大程度减少不必要的医疗干预，改善患者生活质量。

## Abstract (en)

With the wide application of low-dose spiral computed tomography and the advancement of imaging technology, the detection rate of pulmonary nodules has significantly increased. However, the traditional "one-size-fits-all" management strategy has been unable to meet the needs of precise diagnosis and treatment. This expert consensus was initiated by Professor Chunxia SU’s team and Professor Chang CHEN’s team from Shanghai Pulmonary Hospital and jointly formulated by experts from multiple disciplines including oncology, respiratory medicine, thoracic surgery, interventional medicine, radiology, and pathology. The aim is to provide scientific and practical decision-making references for clinical controversies that are less addressed or have differences in existing guidelines. The consensus uses the GRADE method for evidence assessment and forms 18 recommendations through two rounds of Delphi questionnaire surveys, focusing on differentiated screening for special populations, artificial intelligence assisted diagnosis, robot-assisted puncture, application of molecular markers, active monitoring of low-risk nodules, timing of invasive intervention, termination conditions of follow-up, management strategies for multiple nodules, and selection of individualized lymph node dissection range and surgical methods for early-stage lung cancer. This consensus would provides supplementary guidance for the entire process of precise management of pulmonary nodules from screening to treatment, with expectation of improving the long-term survival rate of lung cancer patients while minimizing unnecessary medical interventions and enhancing the quality of life of patients.

随着低剂量螺旋计算机断层扫描（low-dose spiral computed tomography, LDCT）筛查的广泛应用及影像技术的进步，肺结节检出率显著提高。不同结节的生物学行为具有显著异质性，传统的“一刀切”管理策略已不能满足现代诊疗需求。精准医学的兴起为肺结节的管理带来革命性变化，肺结节筛查、诊断、随访及治疗各阶段中众多争议焦点问题日益突出。本专家共识不以制定肺结节管理路径为目标，而聚焦现有指南或专家共识关注较少、存在分歧，且临床实践中争议较大的临床问题，主要包括特殊人群的差异化筛查、人工智能（artificial intelligence, AI）辅助诊断、机器人辅助穿刺、分子标志物的应用、低风险结节的主动监测、侵入性干预、随访终止的时机、多发结节的管理策略以及早期肺癌个体化淋巴结清扫范围和手术方式的选择等，向国内肿瘤内科、呼吸内科、胸外科、介入科、影像科、病理科等多学科专家征询意见，旨在为临床医生提供科学、实用的决策参考，在平衡筛查获益与过度诊疗风险、精准识别高危人群、合理应用新技术以及优化个体化治疗方案等方面提出专家建议，以期在提升肺癌患者长期生存率的同时，最大程度地减少不必要的医疗干预，改善患者的生活质量。

## 1 共识制定背景

### 1.1 肺癌流行病学及肺结节筛查现状

基于全球疾病负担研究（Global Burden of Disease Study, GBD）2022数据^[1]^，肺癌仍然是全球发病率和死亡率均居首位的癌症。2022年，全球新发肺癌病例近250万例，死亡病例超过180万例，分别占全球癌症总发病的12.4%和总死亡的18.7%。而中国是全球肺癌疾病负担最重的国家，发病和死亡人数均占全球发病和死亡人数的40%以上：新发106.1万例，死亡73.3万例。基于人口增长和老龄化的趋势，预测到2050年全球肺癌新发病例将增至462万例，死亡病例将增至355万例；中国新发病例将增至179.5万例，死亡病例将增至140.8万例。因此，我国肺癌防治工作任重道远，在发展新型肺癌诊疗技术的同时，需重点布局全人群的癌症早筛早诊工作，将肺癌的防控线提前至“未病”阶段，从而降低肺癌负担、提高人群健康指数。

#### 1.1.1 筛查技术与标准

当前国内外权威指南，如美国预防服务工作组（The U.S. Preventive Services Task Force, USPSTF）、中国肺癌筛查与早诊早治指南，仍然将LDCT作为肺癌筛查的一线手段。目前，CT设备普遍实现了亚毫米级薄层扫描（≤1 mm），可以对更细微的血管穿行、毛刺征以及磨玻璃成分的变化进行显影，大幅提高了早期肺癌（尤其是原位癌和微浸润癌）的检出率。

同时液体活检辅助肺结节筛查技术正在快速发展，临床已开始引入肺癌抗体七项或循环肿瘤DNA（circulating tumor DNA, ctDNA）甲基化检测等液态生物标志物对影像学难以判定的中高危结节进行辅助诊断。

#### 1.1.2 筛查人群的演变

由于体检意识的增强和CT设备的普及，大量非高危人群被查出肺结节，例如临床发现不吸烟的女性肺结节检出率显著上升，增加了全人群对肺结节筛查的焦虑。研究也开始将筛查人群从“高危”向“泛人群”扩散，对传统“一刀切”的筛查策略提出了新的挑战。

#### 1.1.3 AI辅助诊断

AI已逐渐被开发应用于肺结节高危人群筛选及风险预测模型建立、肺结节影像阅片定性、随访策略制定等肺结节管理各个流程。国内很多三级医院及体检中心已常规配备AI读片系统。但是，目前存在模型多样、缺乏不同人群间的交叉验证等问题。此外，AI影像阅片极高的敏感性导致“假阳性”结节被大量标记，增加了受检者的焦虑感和“过度”的外科治疗。因此，AI辅助肺结节筛查诊断在临床实践中的应用还存在较多问题。

#### 1.1.4 结节管理的精准化

筛查的最终目的是区分“无需处理的良性结节”与“需要干预的早期肺癌”。目前的管理模式趋于精细化，肺结节的风险分层从仅考虑结节大小转变为对影像学特征、生长动力学和临床风险因素的综合评估。治疗管理则越来越强调不同人群的差异化方案。此外，多学科诊疗（multi-disciplinary team, MDT）开始前置化，对于性质难以判定的结节，如混合磨玻璃影（ground glass opacity, GGO）、多发肺结节等，很多大型医院建立了呼吸科、胸外科、影像科的联合门诊，避免患者在不同科室间反复奔波甚至获得矛盾的建议。

#### 1.1.5 面临的挑战与争议

尽管筛查技术先进，但在目前健康管理实践中仍存在以下主要矛盾：（1）过度诊断与过度治疗：大量筛查出的结节是惰性生长的GGO，这类结节可能10年甚至20年都没有实质性进展，属于懒癌或癌前病变。由于公众的恐癌心理，部分本应只需随访的结节接受了外科手术切除，造成了不必要的肺功能损失和医疗资源浪费。（2）辐射与医疗资源平衡：虽然LDCT辐射剂量较低（0.5-1.5 mSv），但针对非高危人群的反复年度筛查，其获益与潜在辐射风险、心理焦虑的比值，依然是公共卫生领域讨论的热点。

### 1.2 肺结节筛查指南及专家共识现状

目前，国内外推出了多部肺结节筛查权威指南，如Fleischner学会指南、美国国立综合癌症网络（National Comprehensive Cancer Network, NCCN）指南、中国肺癌筛查与早诊早治指南等，均对肺结节筛查高危人群、筛查技术标准与质控、结节分类与风险分层、管理路径和随访策略作了详细具体的意见指导。此外，也涌现出越来越多的专家共识，比如《影像学引导下肺结节冷冻消融专家共识（2022版）》^[2]^、《肺部结节（≤2 cm）楔形切除胸外科全国专家共识（2023版）》^[3]^、《肺结节中西医结合全程管理专家共识》^[4]^、《人工智能在肺结节诊治中的应用专家共识（2022年版）》^[5]^等，分别侧重不同层面对肺结节的管理做了更多精细化的指导。

### 1.3 健康管理实践中存在的争议问题

在临床实际工作中，肺癌筛查与诊疗面临着诸多操作层面的争议与困惑，主要体现在以下几个方面。

#### 1.3.1 筛查人群与技术的选择困境

对于不符合传统高危特征的人群，是否应推荐LDCT筛查尚存争议；对于从不吸烟的亚裔女性这一特殊高发群体，如何界定筛查范围以避免过度诊断和资源浪费，缺乏细化标准；同时，对于无法或不愿接受LDCT的人群，血液生物标志物能否作为替代筛查策略，现有证据尚不充分。

#### 1.3.2 新技术应用的角色与边界模糊

随着AI辅助诊断系统和机器人辅助穿刺技术（经皮/支气管镜）的快速发展，其在肺结节全流程管理（如高危人群筛选、影像判读、风险预测、精准活检）中的应用潜力巨大，但不同AI模型性能差异较大，缺乏交叉验证，机器人技术成本高昂且适应证未受到严格把控，临床实践中如何定位这些新技术的角色尚未达成共识。

#### 1.3.3 侵入性干预与随访策略的决策难题

对新发结节和低风险结节的主动监测和侵入性诊断干预时机的选择、对长期稳定的结节安全终止随访条件的把控、多发肺结节风险评估和随访策略的制定等问题，仍是重大挑战。

#### 1.3.4 早期肺癌个体化手术策略的争议

具有不同病理特征的早期肺癌的手术方式选择对于计划亚肺叶切除的早期实性肺癌，术中冰冻提示低分化时是否应立即升级为肺叶切除以及如何通过风险分层实现个体化淋巴结清扫以平衡肿瘤学安全性与手术创伤，这些问题均直接影响患者的远期预后与生活质量。

#### 1.3.5 特殊人群与新技术整合的考量

对于高龄（≥80岁）早期肺癌患者，手术与立体定向放疗（stereotactic body radiation therapy, SBRT）的疗效与安全性需要权衡。基于ctDNA的分子残留病灶（molecular residual disease, MRD）检测在指导术后辅助治疗方面的具体应用仍是临床研究的热点与争议点。

综上所述，为了应对上述从筛查到治疗全过程中存在的争议，本共识工作组整合最新的人群数据及循证医学证据，基于多学科专家经验，形成了针对性的推荐意见，期望在现有指南和专家共识的基础上，对肺结节的管理提供补充和具体的指导意见（图1）。

**图 1:**
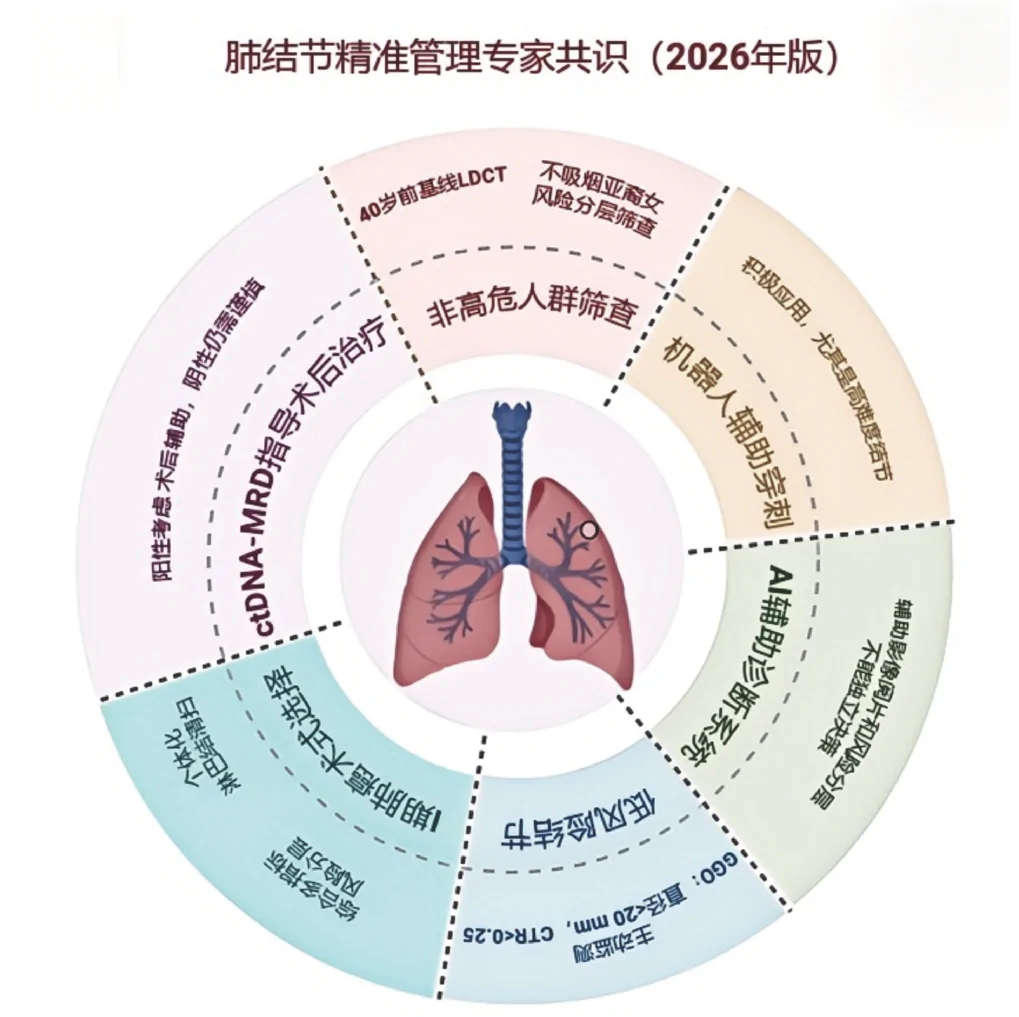
本专家共识主要亮点总结图示 LDCT：低剂量螺旋计算机断层扫描；ctDNA-MRD：循环肿瘤DNA分子残留病灶；GGO：磨玻璃影；CTR：实性成分占比。

## 2 共识形成方法

本共识的报告和撰写参考卫生保健实践指南的报告条目。共识推荐意见的形成结合了推荐分级的评估、制定与评价系统，并综合考量了已发表的专家共识、指南以及高质量的实证研究证据。对于缺乏充分循证医学证据的情况，推荐意见基于专家组的临床经验和独立判断，经专家委员会充分讨论及审查后确定。

### 2.1 共识工作组

本共识由上海市肺科医院苏春霞教授团队和陈昶教授团队共同发起，组织肿瘤内科、呼吸内科、胸外科、介入科、影像科、病理科等相关领域的国内多学科专家共同完成；工作组具体分为3个小组：指导委员会、秘书组和专家组。

### 2.2 共识使用者与目标应用人群

本共识适用人群主要为健康管理科的医务人员、健康管理师以及肺结节管理相关学科的医务人员，如呼吸科、胸外科、放射科、肿瘤科等。本共识目标人群主要为健康体检人群中的肺癌高风险人群及检出肺结节需进一步管理的个体。

### 2.3 临床问题遴选和确定

通过系统检索肺结节及肺癌领域已发表的指南、共识和系统评价，由秘书组初步拟定、专家组讨论、指导委员会审核，最终确定18个临床痛点问题，并根据支持性证据，提出2-3项意见选项。

### 2.4 证据检索

（1）检索数据来源：针对最终纳入的临床问题，检索PubMed、Embase、Cochrane Library、中国生物医学文献数据库、万方和中国知网等数据库，纳入荟萃分析、系统评价以及随机对照试验、队列研究、病例对照研究等，检索时间为建库至2025年12月31日；（2）检索策略：根据前期筛选的临床问题确定相应的检索策略。

### 2.5 证据评价

工具：AMSTAR 2（系统评价）、Jadad量表（随机对照研究）、纽卡斯尔-渥太华（Newcastle-Ottawa Scale, NOS）量表（非随机对照研究）、QUADAS-2（诊断试验）。分级：采用GRADE方法将证据质量分为高（A）、中（B）、低（C）、极低（D）。综合评估框架见表1。

**表1:** 证据等级及共识水平

类别	评价标准
共识水平	
1	专家组就诊疗措施的适宜性达成共识（同意比例>80%）
2	专家组基本就诊疗措施的适宜性达成共识（同意比例50%-80%）
3	专家组存在重大分歧（不同意比例>50%）
证据等级	
高（A）	非常有把握观察值接近真实值；进一步研究非常不可能改变我们对效应估计值的信心
中（B）	对观察值有中等把握：观察值有可能接近真实值，但也有可能差别很大；进一步研究很可能对效应估计值的信心产生重要影响，并可能改变估计值
低（C）	对观察值的把握有限：观察值与真实值可能有很大差别；进一步研究非常可能对效应估计值的信心产生重要影响，并很可能改变估计值
极低（D）	对观察值几乎没有把握：观察值与真实值可能有极大差别；任何效应估计值都是非常不确定的

### 2.6 推荐意见形成

采用德尔菲法问卷调查征集专家意见，经过2轮投票，对推荐意见达成专家共识，最终形成18条推荐意见。

### 2.7 专家共识更新

预计每3-5年对专家共识进行更新。

### 2.8 传播和实施

本共识发布后，将主要通过以下方式进行传播与推广：（1）在相关学术期刊发表；（2）在国内外不同区域、不同学科组织专家共识推广专场，确保临床专业人员充分了解并正确应用本专家共识；（3）通过网络和其他媒体进行推广。

### 2.9 共识注册

本共识已在国际实践指南注册与透明化平台（http://guidelines-registry.org）注册（注册号：PREPARE-2026CN809）。

### 2.10 利益冲突说明

本共识工作组成员均填写了利益冲突声明表，不存在与本共识内容直接相关的利益冲突。

## 3 共识内容

### 3.1 对于不符合高危特征的人群，是否推荐在健康查体中常规进行LDCT的筛查

专家共识1：推荐在40岁前进行一次基线LDCT，但需根据筛查结果及个体危险因素（尤其是年龄、吸烟史、家族史、职业暴露）制定后续随访计划。对于无任何已知危险因素的个体，应充分沟通过度诊断风险后，由医患共同决策（1，B）。

广州爱肺计划针对40岁以上全人群的LDCT筛查结果^[6]^显示，所有11,708例参与者中，仅有1883例（16.1%）和4902例（41.9%）分别符合NCCN 2022标准（年龄≥50岁且吸烟史≥20包年）和中国高危人群标准，而在诊断肺癌的病例中仅有38例（19.0%）和112例（56.0%）符合NCCN标准和中国高危人群标准。换言之，NCCN 2022标准和中国高危人群标准的漏诊率分别为81.0%（n=162）和44.0%（n=88）。即使对于有吸烟史的患者，仅凭高危标准筛查，无法全面覆盖所有需要筛查的人群。美国的一项2017年发表的研究^[7]^提示，按USPTF 2013标准（年龄50-80岁，吸烟史≥30包年，且目前吸烟或戒烟≤15年），1046例曾经吸烟者并且诊断为肺癌的患者中，40%的吸烟者没有达到年龄的要求，20%的吸烟者没有达到30包年以上，30%的吸烟者在诊断前15年即戒烟，综合来看按照高危人群标准至少漏掉50%需要筛查的人群。TALENT研究^[8]^显示从不吸烟者的肺癌检出率为2.6%，远超过NLST研究中的1.1%和NELSON研究中的0.9%，而且有家族史者检出率3.2%，无家族史2.0%，多发家族史达7.2%。基于中国的肺癌发病流行病学特征，中国专家提出了针对低危人群应当采取折中的筛查策略，建议将第1次基线LDCT的时间提前至30岁附近，并根据基线CT的发现结果，结合不同年龄段和其他危险因素将随访间隔延长2-10年^[9]^。对1990-2020年中国及全球肺癌流行病学的数据分析发现：不论男性还是女性，40岁以下的发病率均较低（<1/10万），40岁之后则迅速上升^[10]^。基于这些证据，共识专家组96.9%的成员同意推荐不符合高危特征的人群进行基线LDCT检查，并根据检查结果进行分层管理。

### 3.2 从不吸烟的亚裔女性群体是否可纳入常规年度肺癌筛查

专家共识2：不推荐对从不吸烟的亚裔女性群体进行无条件普筛。该群体内部风险存在异质性。为避免普筛导致的过度诊断和资源浪费，应进行风险细分。建议参照中国指南高危人群定义，仅对年龄>40岁且合并至少一项额外高危因素的个体提供筛查（1，B）。

亚裔肺癌患者的流行病学特征与西方患者具有显著差异。美籍华裔从不吸烟女性肺癌发病率为22.8/10万，菲律宾裔为20.1/10万，其他亚裔为20.3/10万，远高于西班牙裔白人（8.5/10万）和夏威夷和太平洋岛原住民（15.2/10万）^[11]^。GBD 2021的数据分析^[12]^显示，中国男性肺癌发病率居高不下，而女性肺癌发病率呈现快速攀升趋势，2015至2021年，中国年轻女性（<50岁）早发肺癌发病率年均增长2.0% 。中国LDCT筛查结果与西方国家显著不同，检出肺癌中年轻、女性、非吸烟者比例更高^[13]^。中国3个地区6家医院员工常规LDCT健康查体的研究^[14]^显示，女性肺癌检出率显著高于男性（2.5% vs 1.3%, P=0.001），非吸烟者肺癌检出率高于吸烟者（2.2% vs 1.4%, P=0.092）。FANSS研究^[15]^纳入40-74岁从不吸烟的亚裔女性患者，LDCT的肺癌检出率达1.5%，如果将GGO≥5 mm定义为筛查阳性，肺癌检出率可能增加至2.5%-2.9%。但基于中国和韩国的人群研究^[16]^显示，在从不吸烟的低风险亚洲女性中存在显著的非致死性肺癌过度诊断，目前尚无随机试验来评估该患者群体中生存获益程度与潜在危害之间的关系，当前预测的死亡率降低均为推测结果。需要指出的是，上述研究均未排除吸烟以外的其他高危因素的影响。事实上，目前中国通行的筛查指南已经对肺癌高危人群的定义进行了扩展，考虑到了肺癌家族史、吸烟相关的其他恶性肿瘤病史、环境或职业暴露或慢性肺部疾病史等高危因素。尽管目前缺乏对比研究来评估具有与不具有上述危险因素的亚洲非吸烟女性人群在肺癌检出率上的差异，但96.9%的专家组成员认为，依据这些危险因素来界定筛查人群，将有助于在亚洲非吸烟女性独特的流行病学特征与总体筛查效益之间达到更优的平衡。

### 3.3 对于肺癌筛查中不适宜或不愿接受LDCT检查的人群，血液生物标志物可否推荐作为替代筛查策略

专家共识3：目前不推荐血液生物标志物作为肺癌筛查的独立替代策略。但对于因禁忌证（如妊娠、不能耐受）或强烈拒绝接受LDCT且属于肺癌高危的个体，在充分知情同意后，可考虑作为研究性或探索性补充手段（1，C）。

在肺癌筛查或早诊中研究的血液生物标志物主要包括基于血液DNA的ctDNA和血液游离DNA（cell-free DNA, cfDNA）；基于血液蛋白质的传统肿瘤标志物、肿瘤相关自身抗体、补体片段、蛋白质组学信息等。有研究^[17]^利用机器学习的方法开发了一个基于ctDNA的肺癌早筛模型Lung-CLiP，该模型在98%特异度的水平下，对腺癌的预测敏感性为44%，非腺癌为74%。基于肿瘤相关自身抗体的经典肺癌早筛模型EarlyCDT-Lung目前包含7种自身抗体，一项纳入1600例高危人群的研究数据^[18]^提示EarlyCDT-Lung阳性人群的肺癌发生率是阴性人群的5.4倍，整体预测特异度可达91%，但灵敏度仅为37%。既往认为血液标志物灵敏度低，用于大范围人群筛查的效益低，但也有些高敏感性的标志物在研究中，如cfDNA片段化模式对肺癌筛查的灵敏度为80%，特异度为50%^[19]^；但均缺乏前瞻性大样本的验证。血液生物标志物检测具有无创、无辐射等优势，随着检测技术与机器学习的进步，用于肺癌筛查的血液生物标志物模型前景可期，但专家成员一致同意（100.0%）现有证据尚不支持其作为肺癌筛查的独立替代策略，但对于因禁忌证（如妊娠、不能耐受）或强烈拒绝接受LDCT且属于肺癌高危的个体，在充分知情同意后，可考虑作为研究性或探索性补充手段。

### 3.4 AI辅助诊断系统在肺结节管理全过程中的应用角色及应用权限如何定位

专家共识4：推荐AI辅助诊断系统作为影像阅片和风险分层的辅助工具，但不推荐其独立出具最终诊断报告。所有AI标记的阳性结节需经具有资质的影像科医师复核确认。鼓励开展基于本地数据的外部验证及前瞻性临床实效研究（1，B）。

随着机器学习算法的不断增进，已经有越来越多的AI辅助诊断系统被开发出来，性能指标也不断得到优化^[20]^。毋庸置疑的是，AI辅助诊断是未来医学发展的必然趋势。在高危人群的识别方面，相较于传统标准或临床模型，基于机器学习的大数据模型可更精准地筛选人群。Lee等^[21]^开发的一种基于胸部X线片识别肺癌筛查候选者的深度学习模型可以在减少符合USPSTF 2021标准的肺癌筛查候选者数量的同时，保持对新发肺癌的检出率和阳性预测值。Lu等^[22]^在PLCO筛查试验数据中开发了一种基于胸部X线图像和常见电子病历数据的深度学习模型CXR-LC，在同等规模的筛查人群中，CXR-LC在PLCO数据集中的敏感性高于美国医疗保险和医疗补助服务中心（Centers for Medicare & Medicaid Services, CMS）资格标准（74.9% vs 63.8%, P=0.012），漏诊病例减少30.7%。决策曲线分析显示，CXR-LC的净获益高于CMS资格标准，且与PLCOM_2012_的获益相似。Mao等^[23]^开发的GPT-4o模型通过纵向CT图像动态分析，预测肺结节恶性的平均准确率为0.88；与放射科医生的手动测量结果相比，其测量结节大小的平均组内相关系数为0.91；6位放射科医生的评估显示，GPT-4o捕捉结节特征变化的能力得分为4.17分（中位数，基于Likert评分，满分5.00分）。对于已检测出肺结节的人群，深度学习模型较传统Mayo/Brock或Lung-RADS模型可更好地鉴别结节的恶性风险程度。基于四川大学华西医院及其医联体12万受试者数据开发的深度学习模型C-Lung-RADS通过融合影像、临床等多模态数据信息，将鉴别高危肺结节的灵敏度提升至87.1%，优于Lung-RADS的63.3%^[24]^。在随访策略制定方面，深度学习模型也可提供重要参考。Wang等^[25]^基于NELSON试验数据开发了一种多视角CNN模型，用于区分消退与非消退的新发中等大小肺结节，结果显示，在特异度>90%（即非消退结节被错误识别为消退结节的比例<10%）的前提下，可避免14%个体的随访CT检查。以上研究显示了AI辅助诊断系统在高危人群筛选、影像阅片定性、肺结节风险预测、随访策略制定中的作用及优势，但是目前存在模型多样，缺乏不同人群间的交叉验证，因此在临床实践中的应用尚受到质疑。

2023年，Mikhael等^[26]^开发的深度学习模型Sybil通过单次LDCT图像预测肺癌风险，在不同数据集1年受试者工作特征曲线下面积（area under the curve, AUC）达到0.86-0.94，6年AUC为0.75-0.81。该模型在具有重度吸烟史的亚洲人群中对肺癌表现出优异的鉴别性能（1年AUC为0.94，6年AUC为0.70），但在从不或轻度吸烟亚组中对肺癌的鉴别性能较差（1年AUC为0.89，6年AUC为0.56）^[27]^。一项来自四川大学华西医院健康体检中心的真实世界研究^[28]^显示，与人工解读相比，AI辅助阅读的阳性筛查率和恶性肺结节诊断概率显著更高（P<0.001），AI扫描在检测恶性肺结节方面明显优于人工扫描（97.2% vs 86.4%, P<0.001），AI阅读组的肺癌检测率显著高于人工阅读组（98.9% vs 90.3%, P<0.001）。在肺结节筛查巨大数据量的情境下，AI影像辅助诊断系统在提高工作效率和避免因人为疲劳导致的漏诊和误诊方面具有明显优势。基于以上现状，对于AI辅助诊断系统是否已有成熟模型可直接参与在“高危人群筛选、影像阅片定性、肺结节风险预测、随访策略制定”各流程?是否可开放AI辅助影像诊断系统对无结节和典型良性结节的有限决策权限?专家组成员之间存在巨大争议，但其中达成一致意见（100.0%）的内容为：在影像阅片和肺结节风险预测方面的已有较为成熟的大数据模型，可用于临床辅助，但AI辅助诊断系统目前只能处于辅助角色，不应具备任何决策权限。鼓励开展基于本地数据的外部验证及前瞻性临床实效研究。

### 3.5 机器人辅助肺结节穿刺新技术在临床实践中的应用建议

专家共识5：推荐积极应用机器人辅助肺结节穿刺新技术，但需严格把握适应证及禁忌证，并建立操作规范流程（1，B）。

目前机器人辅助肺穿刺技术主要分为经皮肺穿刺机器人和支气管镜机器人，中国多家医疗中心已经开展机器人辅助肺穿刺技术的临床应用。经皮肺穿刺机器人主要用于辅助CT引导下经皮肺穿刺组织活检、消融治疗、胸腔镜手术术前定位，适应证范围包括普通CT引导下穿刺术的常规情形，同时可扩展至徒手穿刺难度或风险较大的患者，尤其是病灶直径较小（<2 cm）或临近血管、支气管、心脏、膈肌等重要结构的病例。绝对禁忌证同普通CT引导下经皮肺穿刺术，以及主管医生判断如肺血管病变或其他原因导致患者不适合穿刺或无法配合操作者^[29]^。在胸腹肿瘤穿刺活检中，机器人导航组的定位成功率显著高于传统穿刺组（84.1% vs 43.7%），调整次数更少（1.51±1.48 vs 3.51±3.05），CT扫描次数更少（4.99±2.11 vs 7.11±3.74）（P<0.05）；且两组在胸部操作定位时间和并发症发生率方面未观察到统计学差异^[30]^。在射频消融治疗中，与传统徒手操作相比，机器人辅助定位的准确性更高，尖端至靶点距离为6 mm，操作次数更少（0 vs 4.5次，P<0.001），不良事件发生率更低（30% vs 70%, P=0.01）^[31]^。机器人辅助CT引导下肺结节定位一次穿刺成功率为100%，定位器与结节之间的中位偏差为6.1 mm，中位定位时间为25.0 min，且操作过程中未报告任何可观察到的并发症^[32]^。支气管镜机器人的适应证包括需病理或病因学诊断的周围型肺部病变，已诊断但需再评估的周围型肺部病变，尤其适用于特殊病例：直径<2 cm的小结节、无支气管征的病变、需经尖锐角度路径到达的病变、需长时间稳定操作的诊疗（如消融、注药）^[33]^。一项中国前瞻性多中心临床研究^[34]^显示，形状感应机器人辅助支气管镜（shape-sensing robotic-assisted bronchoscopy, ssRAB）操作成功率达100%，其中60%以上肺结节分布在传统技术难以到达的肺外周1/3处，总体诊断率为87.8%，即使在≤2 cm肺结节大小的亚组中，诊断率也达85.7%，在安全性方面，仅有1例无需置管的气胸发生。与虚拟支气管镜导航（virtual bronchoscopic navigation, VBN）相比，ssRAB对诊断周围型肺结节的整体诊断率显著更高（69.0% vs 90.0%, P=0.045），且并发症更少^[35]^。基于以上证据，93.9%的专家同意推荐在临床中积极应用机器人辅助肺穿刺技术，但该技术现阶段存在的呼吸运动跟踪与补偿的复杂性、导航系统的局限性以及力触觉反馈缺失的难题也需要引起重视。建议应用时严格把握适应证及禁忌证，并建立适合本中心的操作规范流程，中国针对机器人辅助经皮肺穿刺以及机器人辅助支气管镜已有了初步专家共识，可进行参考。

### 3.6 对于符合高危标准的人群，经LDCT首次筛查结果阴性后，是否可延长随访间隔

专家共识6：基于传统指南，推荐继续维持每年1次的LDCT随访，但专家组内部对此存在显著分歧（3，C）。

各大临床试验中关于“筛查结果阴性”的定义具有较大差异：NLST试验定义为无结节或非钙化结节（non-calcified nodules, NCN）<4 mm^[36]^；NELSON试验中的阴性结节包括具有良性特征的脂肪/良性钙化结节、体积<50 mm³的实性结节、最小直径<5 mm的胸膜旁实性结节、含非实性成分<8 mm的部分实性结节以及直径<8 mm的非实性结节^[37]^。MILD试验定义为无结节或NCN体积<60 mm³者^[38]^。

因此，美国放射学会于2014年引入的肺部影像报告和数据系统（Lung Imaging Reporting and Data System, Lung-RADS），旨在为肺癌筛查LDCT报告建立一个共同的描述语言和标准化的管理框架，以提高筛查质量。Lung-RADS 将1和2类结节视为“阴性结果”^[39]^。其中Lung-RADS 1类为无结节或具有良性征象的肺结节：完全钙化、中央型钙化、爆米花钙化、同心圆钙化或含脂肪结节。Lung-RADS 2类则包括胸膜旁结节：基线筛查或新发结节直径<10 mm（体积<524 mm³）且实性、边缘光滑，形状为椭圆形、双凸形或三角形；实性结节：直径<6 mm（体积<113 mm³）或新发结节直径<4 mm（体积<34 mm³）；部分实性结节：基线筛查总直径<6 mm（体积<113 mm³）；非实性结节：基线筛查、新发/增长直径<30 mm（体积<14,137 mm³）或直径≥30 mm（体积≥14,137 mm³）稳定或缓慢增长；气管结节：基线筛查、新发或稳定存在的亚段内结节。历经2019和2022年的两次更新，目前LUNG-RADS v2022对肺结节良恶性的鉴别特异度达到85%，灵敏度达到92%^[40]^，已成为临床实践中肺结节评估的主流标准，但相关应用研究多集中于欧美人群，该系统其他地区人群（特别是中国以GGO为主的人群特征）中的表现仍需大量本土研究来验证。《中国肺癌低剂量CT筛查指南（2025年版）》专家共识中将阴性结果定义为直径<6 mm的实性结节或部分实性结节，以及直径<8 mm的非实性结节^[41]^。综上，关于“筛查阴性结果”目前缺乏全球统一标准，不同地区的人群可参考本地区适用的指南定义。

关于“LDCT首次筛查结果阴性后，是否可延长随访间隔”，不同研究的结果不尽相同。在NLST试验数据基础上，利用癌症干预和监测建模网络（Cancer Intervention and Surveillance Modeling Network, CISNET）建立的4种肺癌自然史模型分析提示年度随访可较双年度随访方案更有效地降低死亡^[42]^。因此USPSTF推荐，无论筛查结果，高危人群均应维持年度随访策略。然而MILD试验直接比较显示年度与双年度筛查未发现死亡率显著差异，但双年度可节约1/3的CT扫描资源^[38]^。NELSON试验结果显示筛查基线结节<27 mm³的结节2年癌症发生率仅为0.5%，显著低于高危结节（>206 mm³）的16.9%^[43]^，但是NELSON试验的第4轮筛查中（间隔2.5年）间期癌的数量比前几轮更多，且IIIb/IV期癌症的比例更高，提示2.5年可能为筛查间隔的延长上限^[44]^。因此，专家组对该问题争议较大，仅47.1%的专家组成员支持继续维持每年1次的LDCT随访。

### 3.7 对于新发现的8-20 mm实性或亚实性肺结节，实施侵入性诊断干预的建议

专家共识7：推荐肺癌风险预测模型为低危和/或正电子发射计算机断层显像（positron emission tomography/computed tomography, PET/CT）阴性的结节，暂缓侵入性诊断干预；肺癌风险预测模型为中危及以上的，进一步行侵入性诊断干预（1，B）。

侵入性诊断干预措施一般指经皮肺穿刺、支气管镜穿刺活检以及胸腔镜手术等为了获取组织以进行病理诊断的创伤性操作，可能造成气胸、出血、感染等风险。因此对侵入性诊断干预的指征需要严格把控，以避免过度诊断及不必要的创伤。NELSON研究^[45]^数据分析显示在接受LDCT肺癌筛查的人群中，每轮筛查5%-7%的人会检测到新实性结节，其中4%的人最终被诊断为肺癌，0.7%的人会检测到新亚实性结节，其中6%的人最终被诊断为恶性病变。Pinsky等^[46]^的研究发现在基线后2次年度筛查检查中2.6%的受试者存在新发实性结节，肺癌风险随着结节大小增加而升高，从<4 mm结节的1.1%升至≥20 mm结节的24.0%。传统的肺癌风险预测模型Mayo Clinic模型和Brock模型纳入了年龄、结节大小、癌症史、位置、毛刺征等临床影像特征，对肺结节良恶性的整体鉴别效能AUC约为0.7，在Mayo模型基础上加入了PET/CT的氟代脱氧葡萄糖（flurodeoxyglucose, FDG）代谢信息的Herder模型对实性结节的预测能力得到显著提升，尤其在高风险组（恶性概率>60%）可靠性最高[比值比（odds ratio, OR）=3.3]^[47]^。Lung-RADS系统对亚实性结节鉴别效能AUC为0.78^[48]^。基于中国人群开发的C-Lung-RADS系统对肺结节良恶性鉴别的AUC达0.918^[24]^。因此90.9%的专家组成员同意基于结节大小、肺癌风险预测模型以及PET/CT的结果进行侵入性诊断干预的判断。

### 3.8 对于临床表现和影像学特征高度提示恶性（如Lung-RADS 4B类及以上或Brock模型风险>65%），但经微创活检（支气管镜或经皮肺穿刺）获得非明确诊断（如不典型细胞、炎症/坏死组织）结果的肺结节，后续的管理策略建议

专家共识8：对于可耐受手术的患者，推荐直接进行外科诊断性切除术，尤其是周围型实性结节。对于手术风险较高的患者，建议在MDT讨论后选择：（1）重复活检，可参考PET/CT选择活检部位；（2）短期（1-3个月）密切随访复查CT；（3）如PET/CT强烈提示恶性，可考虑手术（1，B）。

支气管镜活检的诊断准确性为79.0%，经皮肺穿刺活检的诊断准确性为73.6%^[49]^。CT引导穿刺活检假阴性率为8%-16%，其中14.6%非特异性良性结果最终证实为恶性^[50]^。ACCP 2013指南明确非诊断性活检结果不能排除恶性可能，建议临床高度怀疑时重复活检，重复活检成功率为54.5%^[51]^。11-20 mm的良恶性实性孤立性肺结节具有相似的CT特征，但在PET/CT上SUVmax值有显著差异^[52]^。PET/CT鉴别良恶性结节的敏感性为64%，特异性为89%，准确性为76%，提示PET/CT可帮助避免侵入性操作，还可为选择穿刺活检部位提供重要依据^[53]^。根据国内及国际指南，影像学提示高度恶性可能的肺结节具有外科手术指征。外科手术切除不仅可以实现诊断与治疗一体化，而且随着微创技术的精进，胸腔镜手术的有效性和安全性都很高^[54]^。因此93.9%的专家同意，对于可耐受手术的患者，推荐直接进行外科诊断性切除术。对于手术风险较高的患者，建议基于MDT讨论，结合患者个人情况，综合评估临床、影像、病理后制定个体化方案，可选择：（1）重复活检，可参考PET/CT选择活检部位；（2）短期（1-3个月）密切随访复查CT；（3）如PET/CT强烈提示恶性，可考虑手术。

### 3.9 对于连续稳定5年的亚实性肺结节，是否可以终止随访

专家共识9：对于直径<10 mm的纯GGO或直径<8 mm的部分实性结节，连续稳定5年后，可考虑延长随访间隔（如每2-3年一次）或终止随访，但需结合患者年龄、预期寿命及个人意愿（1，B）。

Fleischner学会2017指南对亚实性肺结节推荐5年的安全随访时间，具有广泛国际共识^[55]^，而长期随访研究显示5年的随访时间可能不足。对GGO超10年的随访数据^[56]^显示，5年随访稳定后，仍有13%的GGO会出现生长，而≥10 mm、存在实性部分和高龄（≥65岁）是显著增大的风险因素。对纯GGO超16年的随访数据^[57]^显示，10年稳定后，仍有3.9%的纯GGO会出现生长，所有生长结节手术证实均为腺癌。对持续存在的亚实性结节的生长特性的研究^[58]^发现：纯GGO≥10 mm，生长风险显著升高；部分实性结节≥8 mm，生长风险显著升高。因此，84.8%的专家认为对于仍存在风险的结节，如直径≥10 mm的纯GGO、直径≥8 mm的部分实性结节，连续稳定5年后需要继续随访，而其余风险低的结节可以参考国际指南在稳定5年后延长随访间隔或终止随访，但需结合患者年龄、预期寿命及个人意愿。

### 3.10 对多发肺结节的随访管理，是否应以优势结节为中心，参考孤立结节的管理策略

专家共识10：推荐所有结节综合评估，可应用AI辅助评估，建议MDT会诊制定管理策略（1，B）。

多发肺结节恶性风险评估是当代胸科医学面临的重大挑战，各权威机构间的管理策略存在分歧。Fleischner 学会和NCCN推荐基于主病灶（最大或最可疑结节）进行管理，但该方法缺乏客观选择标准，观察者间变异性高，约20%病例中最大结节并非恶性结节。《肺结节诊治中国专家共识（2024年版）》则建议单独评估每个结节，并建议应用AI和人机MDT评估，对要求个体化诊疗者辅以循环染色体异常细胞（circulating genetically abnormal cells, CAC）或PET/CT评估^[59]^。NELSON研究^[60]^发现肺癌概率并不随结节数量显著改变，支持独立评估每个结节的理念。2025年中国的一项研究^[61]^发现，超多发磨玻璃结节（ground-glass nodules, GGNs）进展风险显著增高，是单发结节的6.64倍，是2-5个结节的3.26倍，且超多发结节中，有33.3%的病例出现了多个结节同步生长的情况。多发肺结节风险评估颇具挑战，现有风险预测模型在单发结节中表现良好，但在多发结节人群中表现未经专门验证^[62]^。AI模型可能具有潜在优势。Chen等^[63]^开发的深度学习模型PKU-M在多发肺结节病例中对良恶性结节显示出优异的区分度，AUC达0.890，高于Brock模型（0.806）、PKU模型（0.780）、Mayo模型（0.739）和VA模型（0.682）；并且该模型比临床医生判断的敏感性提高了14.3%，特异性提高了7.8%。基于以上现状，专家组成员一致同意（100.0%）对每个结节进行独立风险评估，并借助AI模型和MDT制定个体化方案，能更精准鉴别多原发癌与转移，避免决策偏差。

### 3.11 对于符合低风险标准的GGO[如直径≤2 cm、实性成分占比（consolidation-to-tumor ratio, CTR）≤0.25]特别是多发结节的患者，应采用主动监测还是早期手术干预

专家共识11：对于符合低风险标准的GGO（如直径≤2 cm、CTR≤0.25）特别是多发结节的患者，推荐进行主动监测，既避免了不必要的手术，又可最大限度保留肺功能，尤其适合多发GGO患者（1，A）。

Yoshino等^[64]^的长期随访研究纳入了314例以GGO为主的周围型肺癌患者，所有患者接受亚肺叶切除（楔形切除或肺段切除），结果显示10年无复发生存（relapse-free survival, RFS）率为98.6%（95%CI: 96.2%-99.5%），总生存（overall survival, OS）率为98.5%（95%CI: 96.1%-99.4%），且仅1例局部复发，证实了亚肺叶切除的长期安全性。Wu等^[65]^的前瞻性多中心试验（ECTOP1021）对406例多发性GGO患者（≥3个GGO，肿瘤直径≤2 cm，CTR≤0.25）进行了主动监测，中位随访35.4个月（IQR：24.2-56.8个月），结果显示5年OS率为100%，其中8.1%（33/406）出现结节进展，1.5%（6/406）出现新发结节，但仅2.0%（8/406）患者需手术干预且术后病理均为IA1期，有力支持主动监测策略的可行性。Zhong等^[66]^的回顾性研究比较了早期手术组（n=210）和监测后手术组（n=69）共279例GGN患者，术后病例确诊为腺癌，结果显示两组长期预后无显著差异（复发率：0.95% vs 1.4%；死亡率：0.5% vs 1.4%；均P>0.05），表明监测后手术未劣于立即手术。基于这些证据，专家组成员一致同意（100.0%）建议对于低风险GGO，尤其是多个GGO的患者，应采用主动监测策略，建议监测方案如下：每6-12个月复查LDCT，当出现以下任一情况时考虑转化干预：（1）实性成分直径增加≥2 mm；（2）体积倍增时间<400 d；（3）新发实性结节。

### 3.12 对于计划亚肺叶切除的≤2 cm的cT1N0M0实性为主周围型肺腺癌，当术中冰冻病理提示低分化特征时，是否应立即升级为肺叶切除，或是完成亚肺叶切除后根据石蜡病理考虑辅助治疗

专家共识12：对于计划亚肺叶切除的≤2 cm的cT1N0M0实性为主周围型肺腺癌，当术中冰冻病理提示低分化特征时，推荐立即升级为肺叶切除（1，B）。

Fu等^[67]^进行的ECTOP-1015多中心前瞻性研究显示，术中冰冻诊断肺腺癌病理亚型的总体符合率为76.1%。国际肺癌研究协会（International Association for the Study of Lung Cancer, IASLC）提出的分级系统是强有力的预后预测工具。Moreira等^[68]^的研究表明，3级低分化肿瘤（含≥20%实体型、微乳头型或复杂腺体成分）的复发风险显著更高，在训练队列中，5年RFS的AUC为0.749，5年OS的AUC为0.787；在测试队列中，RFS的AUC为0.690，OS的AUC为0.743。在手术方式选择方面，Huang等^[69]^的大型回顾性研究（n=3335）表明，对于病理I期肺腺癌，含有贴壁成分（高分化特征）是独立的良好预后因素，多变量分析显示RFS的风险比（hazard ratio, HR）为0.622（95%CI: 0.491-0.789, P<0.001），OS的HR为0.710（95%CI: 0.534-0.944, P=0.019），间接支持了低分化肿瘤需要更积极治疗的观点。Zhang等^[70]^的研究显示，被术中冰冻病理低估（即术中病理诊断为惰性，但术后石蜡病理证实为浸润性腺癌）的患者大多为中高分化型，而具有低分化特征者极少，该部分患者预后极佳，5年RFS率和5年OS率均为100%，反证了若冰冻明确提示低分化特征时其风险的真实性。综上，低分化意味着高侵袭性和复发转移风险，亚肺叶切除无法进行系统性淋巴结清扫，可能导致分期不足和局部控制不充分，90.9%的专家组成员同意推荐立即升级为肺叶切除。

### 3.13 对于cT1N0肺腺癌患者，是否可采用基于术前风险分层的个体化淋巴结清扫策略平衡肿瘤学安全性和手术创伤

专家共识13：对于cT1N0肺腺癌患者，推荐采用风险分层策略，避免低危患者的过度治疗（1，A）。

Zhang等^[71]^进行的ECTOP-1009多中心随机非劣效性III期试验为省略纵隔清扫提供了高质量证据，该研究将302例CTR≤0.5的周围型肺腺癌患者随机分组，中期分析显示两组均未发现纵隔淋巴结转移，且无清扫组在手术时间（74 vs 109 min, P<0.001）、出血量（44 vs 82 mL, P=0.033）和术后住院时间（3.9 vs 4.5 d, P=0.002）方面显著获益，乳糜胸发生率更低（0% vs 0.7%）。Hattori等^[72]^的回顾性研究聚焦于cT1期纯实性肺癌患者，结果发现叶特异性清扫与系统性清扫的5年OS率无显著差异（72.7% vs 74.3%, P=0.712），经倾向评分匹配后结论依然成立（79.9% vs 78.8%, P=0.665）。在并发症方面，Kamtam等^[73]^的巢式病例对照研究分析33例乳糜胸患者和99例匹配对照，结果显示更侵略性的淋巴结管理（如系统性清扫）与乳糜胸风险增加有关（85% vs 52%, P=0.002），并导致住院时间延长（7 vs 4 d, P=0.003）和再手术率更高（18% vs 1%, P=0.006）。因此，专家组成员一致（100.0%）认为风险分层策略能更精准平衡疗效与创伤。

### 3.14 对于以磨玻璃为主的临床IA期肺腺癌患者，CTR≤0.5是否足以作为省略系统性纵隔淋巴结清扫的单一术前标准

专家共识14：对于以磨玻璃为主的临床IA期肺腺癌患者，推荐综合CTR、生长特性、节段位置、病理类型以及是否有内脏胸膜侵犯或肺门淋巴结转移等多指标进行更精准的风险评估（1，B）。

早期肺腺癌的淋巴结转移预测是指导手术治疗策略的关键。近年来，多项研究基于CT形态学特征、代谢指标和生长特性等方面进行了深入探讨。Zhang等^[71]^进行的一项多中心、开放标签、III期非劣效性随机对照试验比较了系统性纵隔淋巴结清扫与否在GGO为主浸润性肺腺癌患者中的效果。该研究纳入302例临床分期为T1N0M0、CTR≤0.5的患者，中期分析显示，两组均未发现淋巴结转移。不清扫组手术时间显著缩短（74 vs 109 min, P<0.001），术中出血量减少（44 vs 82 mL, P=0.033），术后住院时间缩短（3.9 vs 4.5 d, P=0.002），且无淋巴结清扫相关并发症。基于此，研究者建议对于CTR≤0.5的GGO为主腺癌，可省略纵隔淋巴结清扫。Xu等^[74]^通过一项两中心回顾性队列研究结合23项研究的meta分析（n=6170）评估了GGO的淋巴结转移率。研究发现，纯GGO的纵隔淋巴结转移率为0%，部分实性GGO（CTR≤0.5）的转移率为0.1%，而CTR>0.5时转移率为30.4%。研究强调CTR作为预测指标的重要性，但未提供OR值或CTR细分数据。Lee等^[75]^的回顾性研究纳入593例临床I期部分实性肺腺癌患者，探讨淋巴结转移的预测因素。多变量分析显示，实性成分大小是独立预测因子（OR=1.064, 95%CI: 1.012-1.120, P=0.015），SUVmax>2.5也是显著预测因素（OR=1.202, 95%CI: 1.005-1.437, P=0.044）。分层分析显示淋巴结转移率随实性成分大小增加：cT1a（≤1 cm）为0%，cT1b（>1-2 cm）为5.5%，cT1c（>2-3 cm）为7.1%，cT2a（>3 cm）为13.6%。这表明实性成分大小在风险评估中具有关键作用。Liu等^[76]^研究了144例早期（IA期）肺腺癌患者的生长特性，包括体积倍增时间（volume doubling time, VDT）和质量倍增时间（mass doubling time, MDT）。研究发现，VDT<307 d预测淋巴结转移的AUC为0.860（敏感性为81.5%，特异性为82.9%），MDT<254 d的AUC为0.848（敏感性为77.8%，特异性为84.6%）。多变量分析确认肿瘤大小是独立预测因子（OR=1.352, 95%CI: 1.141-1.584, P<0.001）。这些结果支持VDT和MDT作为术前评估淋巴结转移的有效工具。Zhang等^[77]^提出了6条标准用于预测淋巴结阴性并最终指导选择性淋巴清扫术：（1）CTR≤0.5的患者不需行纵隔淋巴结清扫；（2）术中诊断为以贴壁型为主的腺癌患者不需行纵隔淋巴结清扫；（3）肿瘤位于肺尖段的患者不需行下纵隔淋巴结清扫；（4）肺门淋巴结和内脏胸膜侵犯均为阴性的上叶肿瘤患者不需行下纵隔淋巴结清扫；（5）肺门淋巴结阴性的左上叶肿瘤患者不需行4L淋巴结清扫术；（6）肺门淋巴结阴性的左基底段肿瘤患者不需行上纵隔淋巴结清扫。在一项前瞻性多中心试验（NCT04527419）中纳入720例符合上述1条或多条标准的cT1N0浸润性肺癌患者，所有患者均接受系统性纵隔淋巴结清扫术，旨在评估假设的选择性淋巴清扫策略的准确性。最终分析结果表明：上述选择性淋巴结清扫策略对淋巴结阴性的预测准确性达100%，提示除了CTR，肿瘤节段位置、病理类型、是否有内脏胸膜侵犯或肺门淋巴结转移均对纵隔淋巴结转移有预测价值。基于以上数据，87.8%的专家组成员同意推荐综合多指标进行评估。

### 3.15 对于可切除非小细胞肺癌（non-small cell lung cancer, NSCLC）患者，机器人辅助胸腔镜手术（robot-assisted thoracoscopic surgery, RATS）的显著成本增加是否被其临床优势所抵消

专家共识15：对于可切除NSCLC患者，目前RATS的边际获益不足以支撑其额外成本。但随着技术的成熟和成本的下降，未来的优势可能会愈发明显（1，B）。

在短期手术结局方面，Jin等^[78]^开展的随机对照试验（RVlob试验）短期结果显示，RATS与电视辅助胸腔镜手术（video-assisted thoracic surgery, VATS）在围手术期安全性方面相当，两组均无围手术期死亡发生，术后并发症发生率也无显著差异（14.6% vs 18.4%, P=0.45），两组在手术时间、术后住院时间、胸腔引流管留置时间等方面均具有可比性。然而，RATS组术中失血量更少[100 (50-100) vs 100 (50-150) mL, P=0.04]，但术后胸腔引流总量更高[830 (550-1130) vs 685 (367.5-1160) mL, P=0.007]。此外，在淋巴结清扫质量方面RATS可能更具优势：RATS组清扫的淋巴结总数[11（8-15）vs 10（8-13）枚，P=0.02]和淋巴结站数[6（5-7）vs 5（4-6）站，P<0.001]均显著高于VATS组。特别是对N1站淋巴结的清扫，RATS组也更多[6（4-8） vs 5（3-7）站，P=0.005]，然而，这种更广泛的清扫并未在短期内转化为更高的淋巴结分期上调率（7.6% vs 12.3%, P=0.23）。在成本效益方面，Patel等^[79]^的RAVAL试验提供了早期数据。该研究发现，术后12周，RATS组的健康效用评分显著高于VATS组[0.85 (0.10) vs 0.80 (0.19), P=0.02]。基于12个月的随访数据，研究发现RATS相对于VATS的增量成本效益比为14,925.62美元/质量调整生命年（95%CI: 6843.69-23,007.56）。但基于中国国内医疗体系的成本效益数据尚未见报道。Kent等^[80]^的多中心研究（PORTaL研究）数据显示，RATS组的中转开胸率为7.41%，低于VATS组的15.66%，且5年OS率优于VATS组（81% vs 73%），但该研究为回顾性数据，结果需谨慎解读。目前关于RATS与VATS最重要的长期肿瘤学结局对比，如OS，仍缺乏来自前瞻性随机对照试验的成熟数据。综上，专家组成员一致（100.0%）认为目前RATS的长期生存获益证据不足，而VATS技术成熟、成本低、可及性强，能让更多患者和医疗机构受益，在当下医疗资源现实情况下，应优先提高VATS覆盖率而非追求边际获益有限的昂贵技术。但是同时专家也指出，随着技术的成熟和成本的下降，未来RATS的优势可能会愈发明显，因此在条件允许且患者自愿的前提下，鼓励开展RATS，从而为技术进步提供临床数据积累。

### 3.16 对于医学上可耐受手术的高龄（≥80岁）早期NSCLC患者，应优先考虑手术长期生存和功能独立性优势，或是SBRT短期安全性和即刻生活质量优势

专家共识16：对于医学上可耐受手术的高龄（≥80岁）早期NSCLC患者，结合患者个人意愿的前提下，推荐优先考虑手术（1，C）。

Ye等^[81]^基于监测、流行病学和最终结果（The Surveillance, Epidemiology, and End Results, SEER）数据库的研究表明，经倾向评分匹配后，接受手术的老年（≥65岁）IB期患者中位OS为44.1个月，显著优于未手术组（具体数据未报告），但该研究未直接比较SBRT，而非手术组可能包含其他治疗方式。Pennathur等^[82]^制定的专家共识指出，高风险患者的手术风险取决于一氧化碳弥散量（diffusion capacity of the lung for carbon monoxide, DLCO）、严重肺病、衰弱等因素，围手术期死亡率可达5%-10%。Watanabe等^[83]^针对≥80岁患者的研究结果显示，SBRT的中位OS为60.0个月，且安全性良好，3级放射性肺炎发生率为3.1%。Swaminath等^[84]^的LUSTRE III期试验针对不可手术患者比较了SBRT与常规放疗（conventional radiotherapy, CRT）的结果，发现3年局部控制率无显著差异（87.6% vs 81.2%），为SBRT的疗效提供了高级别证据，但主要针对不可手术人群。在直接比较手术与SBRT的研究中，Park等^[85]^的一项回顾性匹配研究显示，对于≥75岁患者，手术组的5年OS率为65.9%，显著高于SBRT组的40.3%（P=0.048），但癌症特异性生存率无显著差异（P=0.089），提示手术的生存优势可能部分源于患者选择偏倚。关于功能依赖性和长期结局，Hirpara等^[86]^引入了“长期依赖性”指标，结果发现SBRT组在短期（前3个月）死亡或护理院入院风险较低（HR=0.55），但长期（1-5年）风险更高（HR=2.13）；手术组短期依赖风险高（如家庭护理使用率高），但长期功能恢复更好，手术组5年“存活且居家”概率为91%，SBRT组为82%。基于以上数据，结合人均寿命逐渐延长的社会现实，90.9%的专家同意结合患者个人意愿的前提下，推荐优先考虑手术。

### 3.17 对于根治术后ctDNA-MRD阳性的I期NSCLC患者，是否应启动辅助治疗以降低复发风险

专家共识17：对于根治术后ctDNA-MRD阳性的I期NSCLC患者，推荐在MDT讨论下，考虑进行辅助治疗，尤其是对于持续阳性的患者，但需告知目前证据等级有限（2，B）。

术后ctDNA检测用于MRD监测在NSCLC患者中的研究已取得显著进展。Xia等^[87]^开展的前瞻性多中心队列研究（LUNGCA-1）显示，术后（术后3天或1个月）ctDNA-MRD阳性是疾病复发的强预测因子，调整后HR为11.1（P<0.001），而且术后辅助治疗可显著延长ctDNA-MRD阳性患者的RFS，却对ctDNA-MRD阴性患者RFS无改善，提示ctDNA-MRD作为术后辅助治疗决策因素的价值。同一队列的进一步动态监测研究^[88]^显示，完成所有根治性治疗（手术+辅助治疗）后如果ctDNA-MRD仍为阳性的患者无一例外全部复发，而对术前ctDNA阳性且携带可靶向突变[如表皮生长因子受体（epidermal growth factor receptor, EGFR）]的患者，辅助酪氨酸激酶抑制剂（tyrosine kinase inhibibors, TKIs）治疗可显著改善RFS（HR=0.01, P=0.005），且ctDNA清除者预后更好，提示MRD状态作为辅助治疗重要参考依据的价值。Jones等^[89]^设计的TROPION-Lung12 III期临床试验正在评估基于ctDNA阳性或高危病理特征的I期NSCLC患者辅助治疗方案，旨在为MRD指导的策略提供高级别证据。基于以上证据，专家组一致认为ctDNA-MRD在预后分层和复发监测中具有重要价值，推荐所有完成根治性治疗的I-III期NSCLC患者进行ctDNA-MRD动态监测，但仅52.9%的专家组成员支持对于根治术后ctDNA-MRD阳性的I期NSCLC患者进行辅助治疗。

### 3.18 根据传统临床病理特征需要术后辅助治疗但ctDNA-MRD阴性的NSCLC患者是否可以豁免辅助治疗

专家共识18：对于根据传统临床病理特征需要术后辅助治疗[如EGFR突变/间变性淋巴瘤激酶（anaplastic lymphoma kinase, ALK）融合的IB期、II-IIIA期]但ctDNA-MRD阴性的NSCLC患者，目前不推荐仅凭MRD阴性豁免辅助治疗。标准辅助治疗仍是常规。强烈建议此类患者入组临床研究（如适应性降阶梯治疗试验）（2，B）。

传统临床病理特征在术后NSCLC风险分层中的价值已得到广泛验证。一项针对1912例I期肺腺癌的研究^[90]^显示，肿瘤大小（HR=1.30, 95%CI: 1.10-1.55, P=0.003）、脏层胸膜侵犯（HR=1.54, 95%CI: 1.09-2.17, P=0.014）、淋巴血管侵犯（HR=1.70, 95%CI: 1.23-2.34, P=0.001）和IASLC分级（3 vs 1级：HR=7.93，95%CI: 2.81-22.4，P<0.001）是术后复发的独立预测因子。具有3-4个高风险特征的患者5年累积复发率为30%，而无高风险特征的患者仅为4%（P<0.001），表明这些特征能有效识别高危人群。一项前瞻性研究（n=88）^[91]^在根治性治疗后的早期NSCLC患者（其中I期占48.9%）中发现在Landmark时间点（治疗后2周-4个月）ctDNA阳性与复发风险显著关联（HR=14.8, P<10^-5^）。Zhang等^[92,93]^开展的前瞻性队列研究纳入261例I-III期NSCLC患者，在根治性术后动态监测ctDNA-MRD，结果显示持续阴性的患者复发风险仅3.2%，长期随访数据显示，纵向MRD监测的阴性预测值为93.2%，提示ctDNA-MRD持续阴性作为临床治愈标志物的价值。基于以上证据，61.8%的专家认为对于根据传统临床病理特征需要术后辅助治疗（即EGFR敏感突变/ALK融合的IB期以及IIA-IIIB期）但ctDNA-MRD阴性的NSCLC患者是否可以豁免辅助治疗还需要大型前瞻性临床研究进行验证，目前仍建议进行常规术后辅助治疗。强烈建议此类患者入组临床研究，如适应性降阶梯治疗试验，以为将来可能的治疗策略改变提高依据。

## 4 未来展望

本共识围绕肺癌筛查、肺结节管理及早期肺癌诊疗中的18项关键临床问题，基于现有最佳证据形成了阶段性推荐意见。然而，共识中多项推荐仍存在证据等级不高（如B、C级）、专家共识率未达完全一致（如部分问题支持率仅52.9%-64.7%）等情况，反映出该领域仍存在大量未满足的临床需求和科学问题。结合共识中明确指出的研究空白与技术发展趋势，未来可在以下几个方面重点突破。

### 4.1 精准化肺癌筛查策略的构建

传统的肺癌筛查策略以年龄和吸烟史为核心高危因素，但随着亚洲不吸烟女性肺癌发病率的快速攀升以及低危人群中肺结节检出率的显著增加，“一刀切”的筛查策略正面临深刻挑战。

未来肺癌筛查策略的演变将呈现以下趋势：（1）筛查人群的精细化分层。基于遗传风险评分、环境暴露因素（如空气污染、二手烟、油烟暴露等）和基线LDCT结果的个体化风险评估模型将逐步取代传统的二元分类。（2）筛查间隔的个体化调整。随着对惰性肺结节自然病程认识的加深，低风险人群的筛查间隔可能延长至2-5年甚至更长，而高风险人群仍需维持年度随访，从而实现筛查资源的最优配置。（3）液体活检等新型技术的序贯应用。对于LDCT筛查阴性但存在其他高危因素的个体，液体活检可作为补充筛查手段；对于LDCT阳性但影像学特征不典型者，液体活检可辅助进一步的风险分层。未来，随着精准医学理念的深入和更多高质量循证证据的积累，肺癌筛查将从“谁该筛查”的简单问答，发展为“何时筛查、如何筛查、筛查后如何管理”的全流程个体化决策体系。

### 4.2 AI辅助决策系统的临床转化与规范化

AI辅助诊断在肺结节检出、风险分层、随访策略制定中展现出巨大潜力，但目前模型多样性、缺乏交叉验证及泛化能力不足是主要障碍。未来方向包括：（1）建立国家级或区域级的肺结节多模态数据库（整合影像、临床、病理及随访数据），统一数据标准，推动AI模型的训练与外部验证；（2）开展基于AI辅助决策的临床实效性随机对照试验，比较AI联合医生对比传统阅片在肺癌早诊率、良性结节手术率、患者预后及医疗成本方面的差异；（3）开发可解释性AI模型，增强临床医生对AI输出的信任度，并制定AI辅助诊断系统的临床应用规范与质控标准。此外，AI在多发肺结节独立风险评估、结节动态生长预测等复杂场景中的应用值得深入探索。

### 4.3 微创诊疗新技术的循证积累与适应证优化

机器人辅助肺穿刺技术（经皮/支气管镜）的快速发展，正在重塑肺结节介入诊疗的技术格局，已被本共识推荐积极应用，但其高昂成本与临床获益之间的平衡尚需更多真实世界数据。未来需开展多中心登记研究，明确机器人技术相较于传统徒手操作在诊断准确率、并发症率、操作时间及患者满意度等方面的增量价值。特别是对于≤2 cm、无支气管征、邻近重要结构的困难结节，应通过随机对照试验或高质量前瞻性队列验证机器人辅助技术的优效性。同时，随着形状感知机器人、电磁导航支气管镜等新一代设备的迭代，其与消融技术（射频消融、微波消融）的融合有望实现诊断治疗一体化，推动肺癌早诊早治向超微创方向发展。

### 4.4 MRD指导的个体化辅助治疗

ctDNA-MRD正在改变早期肺癌术后管理的范式，但本共识中对于I期MRD阳性患者是否启动辅助治疗、对于传统高风险但MRD阴性患者是否豁免辅助治疗，专家意见尚存分歧。推动MRD指导术后管理策略的临床落地需在以下方面实现突破：（1）检测技术的标准化（包括检测panel、阈值设定、监测频率）和成本优化，目前不同MRD检测平台的技术路径和灵敏度差异较大，统一的技术标准和临床验证将推动MRD的常规临床应用；（2）MRD指导的适应性治疗策略的建立，根据MRD动态变化实时调整辅助治疗方案，实现真正的动态精准管理；（3）MRD与病理完全缓解、影像学评估等多维度指标的整合，构建更加精准的预后预测模型，指导新辅助和围手术期治疗的个体化决策。

### 4.5 全程管理的智能化与闭环化

肺结节管理的终极目标是实现从筛查到诊断、治疗、随访的全流程闭环管理。这一目标的实现依赖于多技术融合和多学科协同。

智能化随访平台将成为未来肺结节管理的重要基础设施。通过AI系统自动整合影像、病理、检验和临床信息，为每个肺结节生成个体化的随访计划，动态监测结节变化并及时预警进展风险，将极大提高管理效率和患者依从性。MDT的常态化与前置化将推动从“被动会诊”到“主动协作”的模式转变——对于性质难以判定的结节，呼吸科、胸外科、影像科、病理科等多学科专家在管理决策的早期即介入讨论，避免患者在不同科室间反复奔波。全程健康管理闭环的构建则是肺结节管理的最终目标——从基线筛查到结节评估、风险分层、随访监测、侵入性干预、术后管理直至安全终止随访，形成完整的管理路径，并通过质控体系确保各环节的规范执行。

## 5 总结

肺癌筛查与早期诊疗已进入一个精准化、智能化、微创化、个体化的新时代。未来10年，随着多组学技术、AI、机器人平台以及液体活检的深度融合，有望实现从“一刀切”式管理向基于风险分层和分子分型的全程精准管理的转变，最终在降低肺癌死亡率与避免过度诊疗之间达到最佳平衡。本共识工作组鼓励各医疗机构和研究者积极参与上述方向的多中心合作研究，共同推动中国肺癌防控事业的高质量发展。

**专家共识编写专家组**

**编写组长：**苏春霞（同济大学附属上海市肺科医院肿瘤综合诊治融合病房）

**编写顾问：**周清华（四川大学华西医院肺癌中心），陈昶（同济大学附属上海市肺科医院胸外科）

**指导专家：**徐嵩（天津医科大学总医院肺部肿瘤外科），闫小龙（空军军医大学唐都医院胸外科），王慧娟（郑州大学附属肿瘤医院/河南省肿瘤医院内科），余宗阳（联勤保障部队第900医院呼吸科）

**编写专家：**周娟（同济大学附属上海市肺科医院肿瘤综合诊治融合病房），张育东（广州医科大学附属第一医院胸外科）

**共识专家组成员**（按姓氏汉语拼音字母排序）：蔡开灿（南方医科大学南方医院胸外科），樊旼（复旦大学附属肿瘤医院放疗科），范江（上海交通大学医学院附属第一人民医院胸外科），方勇（浙江大学医学院附属邵逸夫医院肿瘤科），胡坚（浙江大学医学院附属第一医院胸外科），胡洁（上海市老年医学中心(复旦大学附属中山医院闵行院区)呼吸科），金时（中国医学科学院肿瘤医院深圳医院肿瘤内科），李际盛（山东大学齐鲁医院肿瘤内科），李敏（中南大学湘雅医院呼吸科），李因涛（山东第一医科大学附属肿瘤医院呼吸内科），李媛（复旦大学附属肿瘤医院病理科），梁乃新（中国医学科学院北京协和医院胸外科），廖永德（华中科技大学同济医学院附属协和医院胸外科），刘雨桃（中国医学科学院北京协和医学院肿瘤医院内科），申鹏（南方医科大学南方医院肿瘤科），唐建（南方医科大学南方医院胸外科），田攀文（四川大学华西医院呼吸与危重症医学科），田涛（西安交通大学第一附属医院肿瘤内科），汪进良（解放军总医院肿瘤医学部肿瘤内科），王佳蕾（复旦大学附属肿瘤医院肿瘤内科），王立峰（南京大学医学院附属鼓楼医院肿瘤科），夏旸（浙江大学医学院附属第二医院呼吸与危重症医学科），谢惠康（同济大学附属上海市肺科医院病理科），刑明亮（空军军医大学唐都医院胸外科），杨萌（中日友好医院呼吸与危重症医学科），杨农（湖南省第二人民医院(湖南省脑科医院)肿瘤中心），张红梅（中国医学科学院肿瘤医院影像诊断科），张永昌（中南大学湘雅医学院附属肿瘤医院肿瘤新药研究病房），张勇（复旦大学附属中山医院呼吸科），赵明芳（中国医科大学附属第一医院肿瘤内科），赵晓刚（同济大学附属上海市肺科医院胸外科），周永新（同济大学附属同济医院胸外科）
